# Longitudinal association between the duration of eye gaze fixation on social information and specific symptoms of neurodevelopmental disorders in children: A large‐scale community‐based cohort study

**DOI:** 10.1002/pcn5.70095

**Published:** 2025-05-19

**Authors:** Masatsugu Orui, Mami Ishikuro, Taku Obara, Aoi Noda, Genki Shinoda, Keiko Murakami, Hirohito Metoki, Masahiro Kikuya, Naoki Nakaya, Tomoko Nishimura, Keiko Tanaka, Yoshihiro Miyake, Atsushi Hozawa, Kenji J. Tsuchiya, Shinichi Kuriyama, The Tohoku Medical Megabank Project Study Group

**Affiliations:** ^1^ International Research Institute of Disaster Science Tohoku University Sendai Japan; ^2^ Tohoku Medical Megabank Organization Tohoku University Sendai Japan; ^3^ Graduate School of Medicine Tohoku University Sendai Japan; ^4^ Tohoku University Hospital Tohoku University Sendai Japan; ^5^ Division of Public Health, Hygiene and Epidemiology Tohoku Medical and Pharmaceutical University Sendai Japan; ^6^ Department of Hygiene and Public Health Teikyo University School of Medicine Tokyo Japan; ^7^ United Graduate School of Child Development The University of Osaka, Kanazawa University, Hamamatsu University School of Medicine, Chiba University, and University of Fukui Suita Japan; ^8^ Research Center for Child Mental Development Hamamatsu University School of Medicine Hamamatsu Japan; ^9^ Department of Epidemiology and Public Health Ehime University Graduate School of Medicine Toon Japan

**Keywords:** ADHD, ASD, eye‐tracking, Gazefinder, longitudinal

## Abstract

**Aim:**

Autism spectrum disorder (ASD) is a neurodevelopmental condition clinically characterized by abnormalities in eye contact during social interactions. Eye‐tracking systems have been used to screen individuals with ASD by capturing atypical eye gaze patterns of diagnostic significance, such as reduced duration of eye gaze fixation on social information. However, most prior studies have focused on the screening accuracy of eye‐tracking systems in children already diagnosed with ASD, with few longitudinal assessments conducted on a large scale. This large‐scale, longitudinal, community‐based study aimed to analyze the association between specific neurodevelopmental symptoms and the duration of eye gaze fixation on social information within a community‐based setting.

**Methods:**

A longitudinal study involving 2101 participants utilized a generalized linear model (GLM) to examine associations between the duration of eye gaze fixation on social information at age 4 years and subscale scores of the Strengths and Difficulties Questionnaire (SDQ) at ages 6–7.

**Results:**

GLM analysis revealed that shorter durations of eye gaze fixation on social information at age 4 years were significantly associated with emotional problems and peer problems, and with hyperactivity/inattention attention‐deficit hyperactivity disorder (ADHD) at ages 6–7 years.

**Conclusion:**

These findings demonstrate the ability to detect not only peer problems characteristic of ASD but also hyperactivity/inattention characteristic of ADHD longitudinally, which it might be related to the comorbidity of ASD and ADHD. This preliminary study highlights the potential for neurodevelopmental screening; however, further research is needed to validate the accuracy of these methods.

## BACKGROUND

Autism spectrum disorder (ASD) is a neurodevelopmental condition characterized by deficits in social communication and interaction, along with restricted, repetitive patterns of behavior, interests, and actions. Atypical gaze patterns are among the diagnostic criteria for ASD.^1^ Eye‐tracking systems have been employed to identify these gaze patterns in children and adolescents with ASD.^2^, ^3^, ^4^, ^5^, ^6^, ^7^, ^8^ Additionally, studies have reported associations between shorter durations of eye gaze fixation on social information and psychiatrist‐diagnosed attention‐deficit hyperactivity disorder (ADHD) in children, characterized by increased gaze variability.^9^, ^10^, ^11^, ^12^ However, most studies have assessed the screening accuracy of eye‐tracking systems in children already diagnosed with ASD or ADHD, with limited large‐scale, community‐based investigations. To our knowledge, only one study has conducted a longitudinal validation of an early‐age eye‐tracking biomarker for ASD.^13^

Gazefinder®, an eye‐tracking system, has been used to measure atypical gaze patterns in individuals with ASD. A review of Gazefinder® studies^14^, ^15^, ^16^, ^17^, ^18^, ^19^, ^20^, ^21^ revealed its ability to distinguish between children with ASD and those with typical development.^14^, ^15^, ^16^ However, no studies have examined specific symptoms of neurodevelopmental disorders beyond ASD. While Gazefinder® reasonably detects features characteristic of ASD,^19^ it may also detect ADHD features, as several studies have reported shorter durations of eye gaze fixation on social information in children diagnosed with ADHD.^9^, ^10^, ^11^, ^12^

This study, based on a large‐scale community cohort, aimed to conduct a longitudinal analysis of the relationships between eye gaze data, measured by Gazefinder® at age 4 years, and subscales of neurodevelopmental screening tools. It further explored whether gaze data at age 4 years could predict subsequent neurodevelopmental problems at ages 6–7 years. We hypothesized that (1) gaze data would be specifically associated with neurodevelopmental scale subscales characteristic of both ASD and ADHD, and (2) gaze data at age 4 years could longitudinally detect neurodevelopmental symptoms of ASD and ADHD at ages 6–7 years. We conducted a preliminary univariate analysis previously^22^ in accordance with these hypotheses. Subsequently, we conducted a more comprehensive analysis that included multivariate analysis in the present study.

## METHODS

### Tohoku Medical Megabank Project Birth and Three‐Generation (TMM BirThree) Cohort Study and participants

The Tohoku Medical Megabank (TMM) aims to provide medical care to address the damage caused by the Great East Japan Earthquake in 2011 and to support health services through personalized medical care and assistance for disaster victims. The BirThree Cohort Study, part of the TMM, recruited pregnant women, their children, partners, and parents from specific regions of Miyagi and Iwate Prefectures, Japan, between July 2013 and March 2017. Details regarding the recruitment process have been described previously.^23^, ^24^

**Figure 1:**
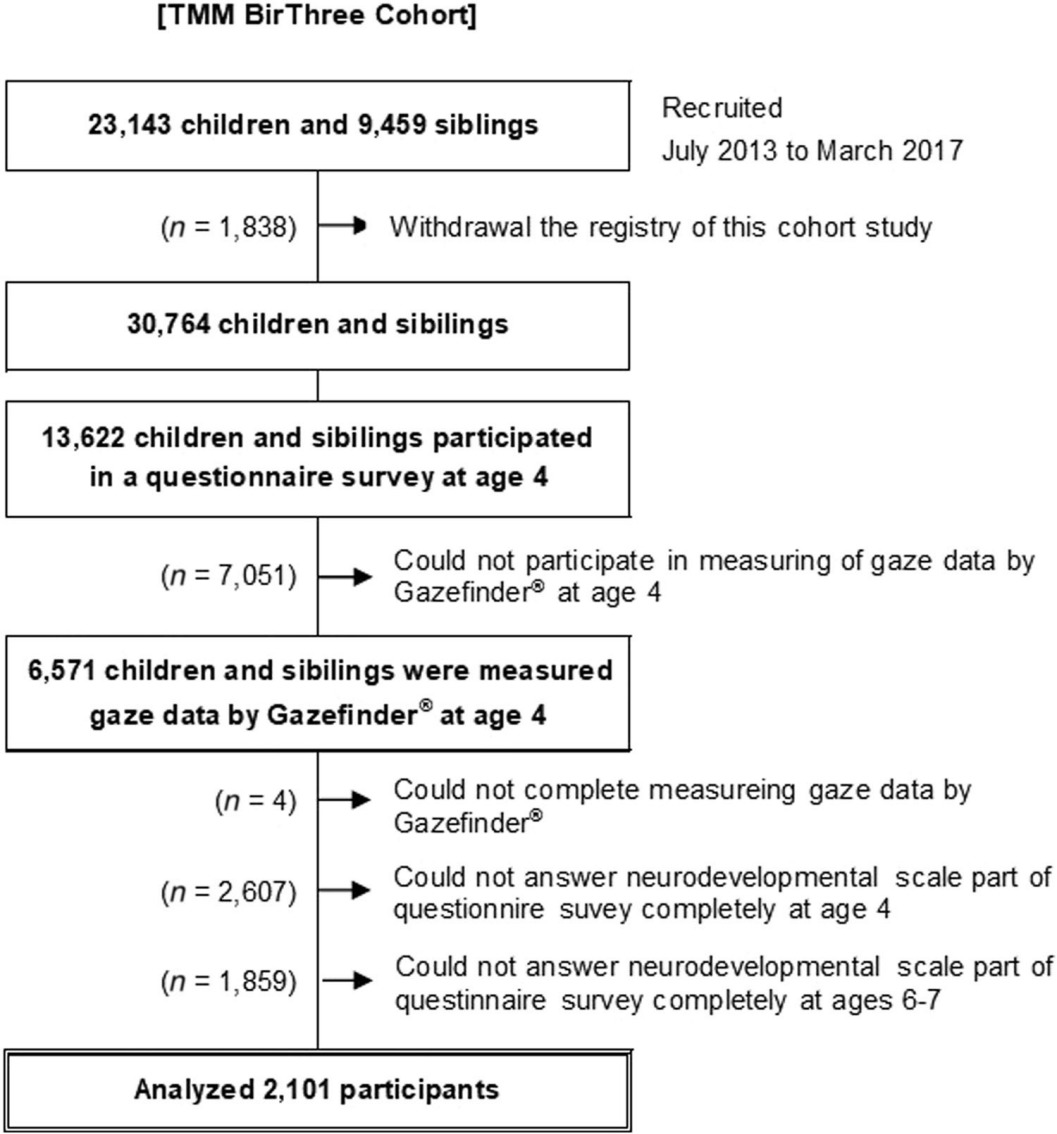
Participants of the Tohoku Medical Megabank Project Birth and Three‐Generation (TMM BirThree) cohort and analyzed subjects.

From 23,143 children and 9459 siblings recruited for the BirThree Cohort Study, 13,622 children and siblings participated in a questionnaire survey at age 4 years and 6571 were measured gaze data by Gazefinder® at age 4 years. Four participants did not complete Gazefinder® measurement of gaze data. Participants who completed the neurodevelopmental scale questionnaire at both ages 4 and 6–7 years and had gaze data available at age 4 years were selected. The final study population consisted of 2101 participants (Figure 1).

The study focused on children at age 4 years because all children in Japan undergo a health checkup at 3 years and 6 months of age. This timeline aligns with the possibility of adopting gaze measurement as part of health checkups in the future.

### Gazefinder® measurements

Participants were seated in front of a monitor for ∼2 min under the supervision of a research coordinator. Eye position was measured using a camera equipped with an infrared light source located below a 19‐inch monitor (1280 × 1024 pixels). Corneal reflection was used to record eye position at a frequency of 50 Hz, capturing *x* and *y* coordinates. Calibration of eye position was performed prior to showing a series of videos. Participants were only required to watch stimuli on the monitor for ∼2 min.

**Figure 2:**
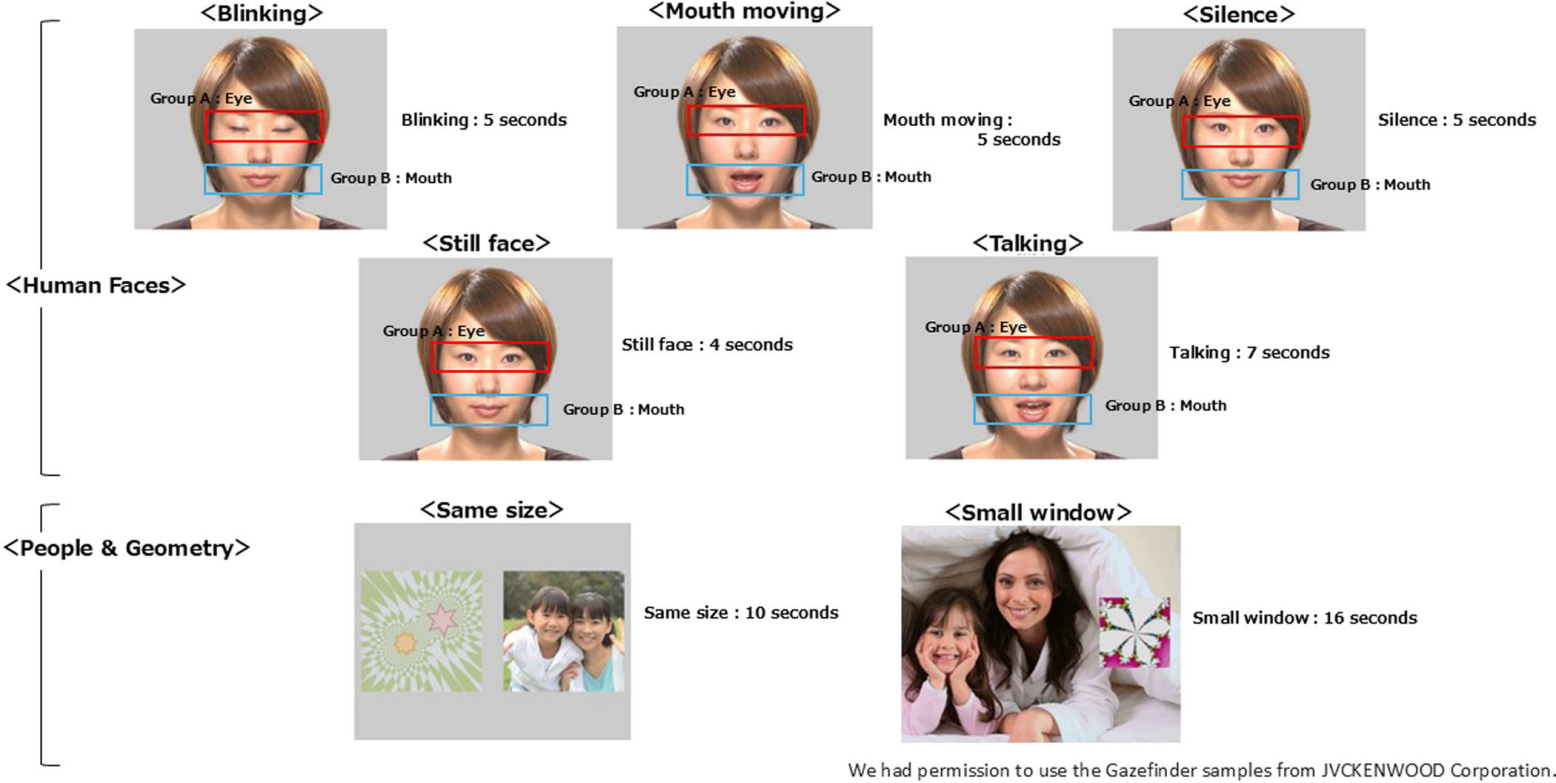
Gazefinder's five stimuli of human faces and two stimuli of people and geometry.

After calibration, eight short videos representing different stimuli were shown. These included five videos featuring human faces with specific expressions or actions: blinking (5 s), mouth moving (5 s), silence (5 s), still face (4 s), and talking (7 s). Additionally, two videos featured people and geometric patterns: same size (images of people and geometric patterns displayed at the same size, 10 s), and small window (geometric patterns displayed in a small frame on an image of people, 16 s) (Figure 2).

Gaze patterns representing social information included gazes directed at the eyes under the various conditions in human face stimuli, and gazes directed at people in the same size and small window conditions of people and geometry stimuli. Social information was therefore defined as gaze patterns directed at eyes in human face stimuli and people in people and geometry stimuli.^14^ The proportion of time spent gazing at these specific objects was measured accordingly.

### Measurement of symptoms of ASD and ADHD using the SDQ at ages 4 and 6–7 years

The Strengths and Difficulty Questionnaire (SDQ) includes 25 questions assessing four domains of difficulties: emotional problems, conduct problems, hyperactivity/inattention, and peer problems. The scale also includes one subscale measuring prosocial behavior. The SDQ is not specific to ASD but serves as a tool for assessing overall mental health in children.^25^, ^26^

The SDQ has demonstrated reliability and validity across various studies and is widely used worldwide for clinical evaluation.^27^ Each of the 25 items is rated on a three‐point scale: not true (0 points), somewhat true (1 point), and certainly true (2 points). Subscale scores range from 0 to 10, with higher scores indicating greater difficulties. For this study, the emotional problems, conduct problems, hyperactivity/inattention, and peer problems subscales were adopted as outcomes.^28^, ^29^ According to a previous study, children diagnosed with ASD had higher median scores on the SDQ's emotional problems and peer problems than children diagnosed with ADHD. Also, children with ADHD diagnoses had high median scores on the SDQ's conduct problems and hyperactivity/inattent.^30^

### Statistical analysis

#### Categorized gaze data at age 4 years

In this study, the duration of eye gaze fixation on the monitor for each stimulus (i.e., “blinking,” “mouth moving,” “silence,” “still face,” and “talking” for human face stimuli, as well as “same size” and “small window” for people and geometry stimuli) was analyzed by referring to a previous study.^14^

First, the distribution of the duration of eye gaze fixation on the monitor for each stimulus was confirmed. There were concerns regarding the reliability of gaze data from children who gazed off‐screen for most of the time. Consequently, children with a duration of eye gaze fixation below the 5th percentile of the whole cohort were excluded from the analysis. Next, the duration of eye gaze fixation for each stimulus was categorized into four groups based on the three quartile points (first quartile, median, and third quartile).

#### Associations between gaze data and neurodevelopmental subscales at age 4 years (first analysis)

In the first analysis, we examined differences in the subscale scores of the SDQ (“emotional problems,” “conduct problems,” “hyperactivity/inattention,” and “peer problems”) at age 4 years after categorizing each stimulus into four groups based on the quartile divisions of gaze data.

To evaluate the differences in SDQ subscale scores for each quartile of gaze data, analysis of covariance (ANCOVA) was performed, adjusted for sex. Subsequently, a generalized linear model (GLM) with gamma distribution was employed.

#### Associations between gaze data and neurodevelopmental subscales at ages 6–7 years (second analysis)

In the second analysis, we examined differences in the subscale scores of the SDQ at ages 6–7 years after categorizing each stimulus into four groups based on the quartile divisions of gaze data at age 4 years.

To evaluate the differences in SDQ subscale scores for each quartile of gaze data at ages 6–7 years, ANCOVA was performed, adjusted for age and sex. Subsequently, GLM with gamma distribution was employed.

#### Overall statistical analysis

All analyses were performed using Stata 18 (StataCorp, 2023. Stata Statistical Software: Release 18. StataCorp LLC).

### Ethical considerations

This study was approved by the ethics committee of the Tohoku University School of Medicine (approval: 2013‐4‐103; date: May 10, 2013; latest revision: 2023‐4‐040; approval date: June 21, 2023). All participants in the TMM BirThree Cohort Study provided informed consent. As all participants in this study were minors, their guardians provided proxy consent. Finally, this study was conducted in compliance with the Declaration of Helsinki, as it involved human participants.

## RESULTS

### Basic characteristics of participants

The number of excluded participants for each stimulus was as follows: blinking (*n* = 112), mouth moving (*n* = 109), silence (*n* = 113), still face (*n* = 109), talking (*n* = 112), same size (*n* = 203), and small window (*n* = 213). The number of male (age 4 years, 1090; age 6 years, 215; age 7 years, 875) and female (age 4 years, 1011; age 6 years, 193; age 7 years, 818) participants was approximately equal. The SDQ score were shown in Table 1. A higher score on the subscales indicates the presence of neurodevelopmental problems. The SDQ subscale scores for conduct problems and hyperactivity/inattention at ages 6–7 years decreased compared to the scores at age 4 years, while the scores for other subscales showed minimal changes.

**Table 1:** SDQ subscale score of participants (score range: 0–10 points).

	First analysis: age 4 years					Second analysis: ages 6–7 years				
	Mean	SD	25% tile	Median	75% tile	Mean	SD	25% tile	Median	75% tile
Emotional problems	1.70	1.72	0.00	1.00	3.00	1.82	1.99	0.00	1.00	3.00
Conduct problems	2.34	1.58	1.00	2.00	3.00	2.09	1.65	1.00	2.00	3.00
Hyperactivity/inattention	3.62	2.16	2.00	3.00	5.00	3.31	2.21	2.00	3.00	5.00
Peer problems	1.72	1.60	0.00	1.00	3.00	1.74	1.62	0.00	1.00	3.00

Abbreviations: SD, standard deviation; SDQ, the Strengths and Difficulty Questionnaire.

### Differences between gaze data and neurodevelopmental subscales at age 4 and ages 6–7 years

In the first analysis, ANCOVA was conducted to assess differences in the SDQ subscale scores at age 4 years across quartile categories of gaze data for each stimulus that age. Among the five human face stimuli (“blinking,” “mouth moving,” “silence,” “still face,” and “talking”), a shorter duration of eye gaze fixation on a person's eye during the “still face” stimulus was significantly associated with higher peer problem scores on the SDQ subscale. For the stimuli involving people and geometry (“same size” and “small window”), a shorter duration of eye gaze fixation on people during the “same size” stimulus was significantly associated with higher scores for hyperactivity/inattention and peer problems on the SDQ subscale. Similarly, a shorter duration of eye gaze fixation on people during the “small window” stimulus was significantly associated with higher scores for emotional problems and hyperactivity/inattention (Tables 2‐1–2‐4).

**Table 2:** Difference of gaze data at age 4 yeras by quartile points and subscale score of SDQ at age 4 years.

	Q1		Q2		Q3		Q4		*p* value
	Mean	SD	Mean	SD	Mean	SD	Mean	SD	Adjusted
Table 2‐1 Emotional problems (score range: 0–10 points)									
*Human images*									
Blinking	1.716	1.616	1.822	1.864	1.683	1.718	1.593	1.626	0.374
Mouth moving	1.686	1.796	1.729	1.727	1.731	1.656	1.707	1.716	0.991
Silence	1.679	1.658	1.872	1.987	1.718	1.620	1.568	1.579	0.163
Still face	1.821	1.857	1.725	1.739	1.643	1.621	1.598	1.663	0.334
Talking	1.748	1.699	1.815	1.834	1.682	1.683	1.577	1.678	0.364
*People and geometry*									
Same size	1.743	1.788	1.731	1.676	1.613	1.660	1.680	1.726	0.658
Small window	1.482	1.604	1.797	1.782	1.842	1.745	1.658	1.697	0.044
								*Adjusted by sex	
Table 2‐2 Conduct problems (score range: 0–10 points)									
*Human images*									
Blinking	2.438	1.599	2.407	1.694	2.243	1.547	2.201	1.453	0.143
Mouth moving	2.466	1.595	2.341	1.509	2.239	1.620	2.278	1.574	0.276
Silence	2.351	1.548	2.324	1.515	2.320	1.538	2.300	1.693	0.984
Still face	2.402	1.552	2.377	1.603	2.274	1.548	2.273	1.633	0.620
Talking	2.421	1.583	2.365	1.616	2.364	1.590	2.184	1.554	0.251
*People and geometry*									
Same size	2.434	1.585	2.456	1.644	2.295	1.575	2.202	1.479	0.179
Small window	2.405	1.546	2.395	1.603	2.294	1.591	2.280	1.562	0.700
								*Adjusted by sex	
Table 2‐3 Hyperactivity/inattention (score range: 0–10 points)									
*Human images*									
Blinking	3.796	2.100	3.685	2.177	3.505	2.147	3.353	2.121	0.050
Mouth moving	3.665	2.148	3.694	2.035	3.431	2.100	3.582	2.269	0.413
Silence	3.757	2.171	3.564	2.088	3.547	2.124	3.447	2.180	0.378
Still face	3.804	2.207	3.590	2.139	3.666	2.146	3.359	2.095	0.054
Talking	3.742	2.015	3.626	2.188	3.599	2.133	3.426	2.287	0.281
*People and geometry*									
Same size	3.852	2.114	3.758	2.176	3.719	2.179	3.217	1.979	0.007
Small window	3.884	2.037	3.778	2.347	3.397	2.015	3.438	2.037	0.018
								*Adjusted by sex	
Table 2‐4 Peer ploblems (score range: 0–10 points)									
*Human images*									
Blinking	1.728	1.552	1.662	1.528	1.751	1.688	1.669	1.559	0.900
Mouth moving	1.723	1.528	1.700	1.575	1.661	1.534	1.743	1.692	0.950
Silence	1.727	1.596	1.829	1.594	1.753	1.593	1.523	1.565	0.066
Still face	1.821	1.564	1.762	1.666	1.758	1.567	1.491	1.512	0.029
Talking	1.757	1.514	1.764	1.643	1.584	1.502	1.735	1.692	0.374
*People and geometry*									
Same size	1.984	1.759	1.918	1.661	1.649	1.554	1.342	1.338	<0.001
Small window	1.946	1.611	1.695	1.710	1.668	1.512	1.539	1.530	0.055
									*Adjusted by sex

Abbreviations: SD, standard deviation; SDQ, the Strengths and Difficulty Questionnaire.

In the second analysis, which examined associations between SDQ subscale scores at ages 6–7 years and gaze data collected at age 4 years, shorter durations of eye gaze fixation on the “still face,” “talking,” “same size,” and “small window” stimuli were associated with various SDQ subscale items. Considering the findings from age 4 years, a shorter duration of eye gaze fixation on people during the “small window” stimulus showed a significant association with peer problems, while the “same size” stimulus was significantly associated with higher scores for hyperactivity/inattention at both age 4 and ages 6–7 years (Tables 3‐1–3‐4).

**Table 3:** Difference of gaze data at age 4 years by quartile points and subscale score of SDQ at ages 6–7 years.

	Q1		Q2		Q3		Q4		*p* value
	Mean	SD	Mean	SD	Mean	SD	Mean	SD	Adjusted
Table 3‐1 Emotional problems (score range: 0–10 points)									
*Human images*									
Blinking	1.802	1.946	1.867	2.086	1.837	2.027	1.764	1.907	0.872
Mouth moving	1.761	1.997	1.770	1.975	1.846	2.036	1.849	1.919	0.840
Silence	1.808	1.961	1.849	2.078	1.918	2.032	1.704	1.897	0.408
Still face	1.815	2.008	1.817	2.017	1.816	1.978	1.803	2.012	0.999
Talking	1.882	2.005	1.833	1.999	1.813	2.027	1.759	1.957	0.801
*People and geometry*									
Same size	1.893	1.996	1.878	2.021	1.661	1.915	1.829	2.035	0.153
Small window	1.932	1.985	1.974	2.127	1.625	1.864	1.742	1.984	0.011
							*Adjusted by sex and age		
Table 3‐2 Conduct problems (score range: 0–10 points)									
*Human images*									
Blinking	2.273	1.732	2.023	1.636	1.979	1.585	2.039	1.621	0.031
Mouth moving	2.180	1.753	2.082	1.680	2.121	1.591	1.954	1.584	0.179
Silence	2.218	1.695	2.074	1.676	2.073	1.603	1.968	1.598	0.131
Still face	2.128	1.634	2.245	1.739	2.022	1.569	1.967	1.628	0.042
Talking	2.199	1.728	2.086	1.602	2.057	1.558	1.970	1.659	0.170
*People and geometry*									
Same size	2.076	1.612	2.207	1.703	2.086	1.690	2.025	1.592	0.498
Small window	2.171	1.629	2.077	1.672	2.042	1.554	2.109	1.750	0.742
							*Adjusted by sex and age		
Table 3‐3 Hyperactivity/inattention (score range: 0–10 points)									
*Human images*									
Blinking	3.500	2.146	3.286	2.165	3.212	2.223	3.165	2.282	0.107
Mouth moving	3.288	2.196	3.363	2.180	3.314	2.205	3.192	2.193	0.612
Silence	3.438	2.169	3.402	2.269	3.224	2.180	3.126	2.177	0.055
Still face	3.457	2.182	3.418	2.181	3.149	2.189	3.236	2.288	0.140
Talking	3.518	2.225	3.301	2.200	3.260	2.161	3.127	2.215	0.045
*People and geometry*									
Same size	3.519	2.244	3.403	2.177	3.314	2.268	3.116	2.115	0.405
Small window	3.672	2.271	3.515	2.308	3.110	2.079	3.058	2.095	<0.001
							*Adjusted by sex and age		
Table 3‐4 Peer problems (score range: 0–10 points)									
*Human images*									
Blinking	1.796	1.601	1.690	1.585	1.706	1.681	1.779	1.579	0.702
Mouth moving	1.798	1.619	1.726	1.631	1.682	1.602	1.763	1.611	0.705
Silence	1.722	1.590	1.799	1.742	1.783	1.551	1.665	1.566	0.469
Still face	1.724	1.663	1.815	1.731	1.863	1.593	1.598	1.498	0.039
Talking	1.779	1.624	1.846	1.628	1.641	1.579	1.722	1.614	0.218
*People and geometry*									
Same size	1.972	1.777	1.906	1.649	1.598	1.476	1.477	1.451	<0.001
Small window	1.835	1.687	1.825	1.661	1.711	1.474	1.566	1.564	0.087
							*Adjusted by sex and age		

Abbreviations: SD, standard deviation; SDQ, the Strengths and Difficulty Questionnaire.

### Associations between gaze data at age 4 years and neurodevelopmental subscales at age 4 and ages 6–7 years

For the first analysis, a GLM with a gamma distribution was applied. The results showed that a shorter duration of eye gaze fixation on social information during the “talking” stimulus was significantly associated with conduct problems at age 4 years (*β* = −0.878, P = 0.016). Additionally, a shorter duration of eye gaze fixation on social information during the “same size” stimulus was significantly associated with peer problems at age 4 years (*β* = −0.879, P = 0.017).

Subsequently, for the second analysis, a GLM with a gamma distribution was performed. The results indicated that a shorter duration of eye gaze fixation on social information during the “talking” stimulus was significantly associated with hyperactivity/inattention at ages 6–7 years (*β* = −0.760, P = 0.049). Furthermore, a shorter duration of eye gaze fixation on social information during the “same size” stimulus was significantly associated with peer problems at ages 6–7 years (*β* = −0.992, P = 0.002). A shorter duration of eye gaze fixation on social information during the “small window” stimulus was significantly associated with emotional problems (*β* = −0.729, P = 0.030) and hyperactivity/inattention at ages 6–7 years (*β* = −1.218, P = 0.001) (Tables 4‐1–4‐4).

**Table 4:** Generalized limear model for association between gaze data at age 4 years and subscale score of SDQ.

	First analysis: age 4 years			Second analysis: ages 6–7 years		
	*β*	95% CI	*p* value	*β*	95% CI	*p* value
Table 4‐1 Emotional problems						
Sex (male: 0, female: 1)	0.188	(−0.026, 0.403)	0.085	0.020	(−0.221, 0.261)	0.870
Age (ages 6 or 7 years)				0.248	(0.048, 0.448)	0.015
*Human images*						
Blinking	−0.342	(−1.035, 0.352)	0.334	−0.168	(−0.764, 0.427)	0.580
Mouth moving	0.236	(−0.662, 1.133)	0.607	0.166	(−0.680, 1.011)	0.701
Silence	−0.328	(−0.892, 0.235)	0.253	0.028	(−0.525, 0.581)	0.920
Still face	−0.206	(−0.813, 0.402)	0.507	0.136	(−0.448, 0.720)	0.649
Talking	−0.341	(−1.069, 0.388)	0.359	−0.427	(−1.124, 0.269)	0.229
*People and geometry*						
Same size	−0.419	(−1.239, 0.401)	0.316	0.375	(−0.350, 1.100)	0.311
Small window	0.284	(−0.542, 1.110)	0.501	−0.729	(−1.386, −0.073)	0.030
Table 4‐2 Conduct problems						
Sex (male: 0, female: 1)	−0.045	(−0.235, 0.145)	0.640	−0.011	(−0.209, 0.187)	0.914
Age (ages 6 or 7 years)				−0.177	(−0.341, −0.014)	0.034
*Human images*						
Blinking	−0.323	(−0.918, 0.273)	0.288	−0.237	(−0.713, 0.239)	0.330
Mouth moving	0.134	(−0.620, 0.888)	0.728	−0.184	(−0.825, 0.457)	0.573
Silence	0.189	(−0.282, 0.661)	0.431	−0.241	(−0.643, 0.160)	0.239
Still face	0.116	(−0.386, 0.618)	0.651	−0.089	(−0.553, 0.374)	0.705
Talking	−0.878	(−1.592, −0.165)	0.016	−0.401	(−0.995, 0.193)	0.186
*People and geometry*						
Same size	−0.263	(−0.984, 0.457)	0.473	0.313	(−0.305, 0.931)	0.320
Small window	−0.071	(−0.735, 0.594)	0.835	−0.078	(−0.638, 0.483)	0.786
Table 4‐3 Hyperactivity/inattention						
Sex (male: 0, female: 1)	−0.413	(−0.667, −0.159)	0.001	−0.003	(−0.265, 0.259)	0.980
Age (ages 6 or 7 years)				−0.676	(−0.896, −0.457)	0.000
*Human images*						
Blinking	0.007	(−0.789, 0.803)	0.987	−0.134	(−0.78, 0.513)	0.686
Mouth moving	0.310	(−0.799, 1.419)	0.584	0.155	(−0.721, 1.031)	0.729
Silence	−0.177	(−0.838, 0.485)	0.601	−0.350	(−0.925, 0.225)	0.233
Still face	−0.298	(−1.001, 0.405)	0.406	−0.035	(−0.642, 0.571)	0.909
Talking	−0.777	(−1.706, 0.153)	0.101	−0.760	(−1.517, −0.003)	0.049
*People and geometry*						
Same size	0.072	(−0.909, 1.054)	0.885	0.601	(−0.241, 1.443)	0.162
Small window	−0.798	(−1.680, 0.084)	0.076	−1.218	(−1.970, −0.467)	0.001
Table 4‐4 Peer problems						
Sex (male: 0, female: 1)	−0.279	(−0.466, −0.093)	0.003	0.028	(−0.164, 0.220)	0.776
Age (ages 6 or 7 years)				−0.106	(−0.267, 0.054)	0.195
*Human images*						
Blinking	0.158	(−0.422, 0.737)	0.594	0.164	(−0.314, 0.642)	0.502
Mouth moving	0.242	(−0.582, 1.066)	0.565	−0.045	(−0.639, 0.548)	0.881
Silence	−0.417	(−0.915, 0.081)	0.101	0.090	(−0.332, 0.512)	0.676
Still face	−0.478	(−1.004, 0.047)	0.075	−0.431	(−0.894, 0.031)	0.068
Talking	0.194	(−0.493, 0.882)	0.579	0.039	(−0.495, 0.572)	0.887
*People and geometry*						
Same size	−0.879	(−1.598, −0.159)	0.017	−0.992	(−1.621, −0.364)	0.002
Small window	−0.259	(−0.900, 0.383)	0.429	−0.232	(−0.778, 0.314)	0.405

*Note*: *β*, coefficient value; 95% CI, 95% confidence interval.

Abbreviations: SD, standard deviation; SDQ, the Strengths and Difficulty Questionnaire.

## DISCUSSION

This large‐scale community‐based study demonstrated a significant association between a shorter duration of eye gaze fixation on social information at age 4 years and the SDQ subscale “peer problems” both at age 4 years and longitudinally at ages 6–7 years. Additionally, a shorter duration of eye gaze fixation on social information at age 4 years was significantly associated with the SDQ subscale “hyperactivity/inattention” at ages 6–7 years, despite no significant association at age 4 years.

We will discuss the SDQ subscales that showed significant associations at age 4 and ages 6–7 years. First, our findings indicated that SDQ peer problems, one of the core features of ASD, were significantly associated with a shorter duration of eye gaze fixation on social information during the “same size” stimulus at both age 4 and ages 6–7 years. These findings align with a previous study, which demonstrated that people and geometry stimuli, such as the “same size” stimulus measured by Gazefinder®.^14^ The reason is assumed that children at risk for an ASD have an unusual preference for geometric repetition since they tend to reduce social attention.^31^ Gazefinder®, as a developed screening tool for identifying atypical gaze patterns in individuals with ASD, is designed to detect the core features of ASD, making these results consistent with its intended purpose.

In addition, hyperactivity/inattention was detected at ages 6–7 years but not at age 4 years in people and geometry stimuli, such as the “small window” stimulus. ASD is often diagnosed before ADHD,^32^ which could explain why “hyperactivity/inattention” was not detected at age 4 years but became relevant at ages 6–7 years. Additionally, another study showed that children with ADHD have showed a developmental delay of executive skills (e.g., planning, working memory, attention, inhibition, self‐monitoring, self‐regulation, and initiation) in comparison with typical development children,^33^ although most preschool children have difficulty performing executive function tasks. Therefore, this could influence our findings because the SDQ's hyperactivity/inattention section includes questions concerning executive functions. Previous studies have shown that the duration of eye gaze fixation on social information, measured by eye‐tracking systems other than Gazefinder®, was associated with psychiatrist‐diagnosed ADHD in children, which might be related that their gaze patterns of children with ADHD had a wider range of gaze movements, relatively shorter fixation times.^34^ This was attributed to increased gaze variability observed in individuals with ADHD.^11^ Another possibility is the comorbidity of ASD and ADHD, as some individuals with ASD often exhibit ADHD symptoms.^35^, ^36^, ^37^, ^38^ Indeed, in a study analyzing two US healthcare databases, ADHD was found to be the most common comorbidity among newly diagnosed individuals with ASD (Medicaid 50.09%, Optum 44.16%).^39^

To further explore the association between ASD–ADHD comorbidity and the duration of eye gaze fixation on social information, we conducted an additional analysis (Table S1). Participants were categorized into three groups based on having SDQ subscale scores above the 95th percentile: (1) hyperactivity/inattention alone, (2) peer problems alone, and (3) both hyperactivity/inattention and peer problems. The analysis revealed no significant association between hyperactivity/inattention alone and a shorter duration of eye gaze fixation on social information. However, participants with symptoms of both hyperactivity/inattention and peer problems exhibited significantly shorter durations of eye gaze fixation during the “small window” stimulus. These findings suggest that the significant association between the “small window” stimulus and hyperactivity/inattention, observed at both age 4 and ages 6–7 years, may be influenced by comorbid cases of ASD and ADHD. However, as this additional analysis was conducted without diagnostic information on ASD and ADHD, further research is necessary to evaluate the relationship between gaze data and ADHD with diagnostic information, as well as ASD, in a community‐based cohort study.

This study has several limitations. First, there may be undiagnosed cases of neurodevelopmental disabilities among the participants. As this cohort study targeted the general population, a certain number of participants were likely undiagnosed or did not report their medical history. Second, there may be respondent bias in the neurodevelopmental scales, such as the SDQ. Parents concerned about developmental delays in their children may provide biased responses on these questionnaires. Third, 4311 parents or guardians did not respond to the neurodevelopmental scale's questionnaires, potentially leading to underestimation or overestimation of the findings. Fourth, the SDQ is not a specific neurodevelopmental scale for ASD or ADHD in children. “Peer problems” are a core symptom of ASD, but several studies have also reported their association with ADHD.^30^, ^40^ Similarly, “hyperactivity/inattention” is a core symptom of ADHD, but has also been reported to be associated with ASD.^30^, ^41^, ^42^ Therefore, further research is necessary to evaluate the relationship between gaze data and diagnostic information of ASD or ADHD. The fifth limitation is representativeness in study area. This study was conducted in a limited area of Japan. Lastly, definitive discrimination methods for neurodevelopmental problems in childhood have not yet been established. With Gazefinder®, the selection and utilization of appropriate stimuli for screening remain under discussion.

Despite these limitations, this study has notable strengths. It targeted community‐based children using a larger sample size than previous studies. Additionally, its longitudinal design provided valuable insights, showing that a shorter duration of eye gaze fixation on social information could help detect specific neurodevelopmental characteristics, such as peer problems and hyperactivity/inattention, several years after the initial measurements.

## CONCLUSION

This large‐scale community‐based study utilizing an eye‐tracking system demonstrated the ability to detect not only peer problems, one of the core features of ASD, but also hyperactivity/inattention, one of the core features of ADHD, longitudinally. As this was a preliminary study of the clinical application of neurodevelopmental screening, further research is necessary to validate the accuracy of screening for ASD and ADHD with diagnostic information.

## AUTHOR CONTRIBUTION

Masatsugu Orui conceptualized and designed this study, conducted the initial analyses, drafted the initial manuscript, and revised the manuscript. Shinichi Kuriyama conceptualized, designed, and organized the BirThree Cohort Study and reviewed the manuscript. Masahiro Kikuya, Hirohito Metoki, Taku Obara, Mami Ishikuro, Keiko Murakami, Aoi Noda, and Genki Shinoda managed the implementation of the BirThree Cohort Study and reviewed the manuscript. Kenji J Tsuchiya, Tomoko Nishimura, Yoshihiro Miyake, Keiko Tanaka, Atsushi Hozawa, and Naoki Nakaya reviewed the manuscript critically. All authors approved the final manuscript as submitted and agreed to be accountable for all aspects of the work.

## THE TOHOKU MEDICAL MEGABANK PROJECT STUDY GROUP

Abe, Hikaru; Abe, Michiaki; Abe, Momoka; Abe, Naomi; Abe, Noriko; Abe, Tomomi; Abe, Yuto; Ahiko, Shizuko; Aiki, Kayo; Aizawa, Hiromi; Akiyama, Yukari; Anzawa, Hayato; Aoki, Eri; Aoki, Yuichi; Arai, Hiroko; Arakawa, Misaki; Asano, Yukie; Baird, Liam; Chiba, Ayano; Chiba, Haruna; Chiba, Ippei; Chiba, Kenji; Chiba, Tetsuo; Endo, Hisako; Fue, Reika; Fujishiro, Futaba; Fujita, Yayoi; Fukunaga, Waka; Funata, Mami; Funayama, Takamitsu; Furuhashi, Sho; Fuse, Nobuo; Fushimi, Junko; Fushiya, Kumiko; Gamo, Tomomi; Gocho, Chinatsu; Gonoi, Katsuhiro; Goto, Maki; Goto, Takahiko; Goto, Yukie; Gouko, Kaori; Haga, Michiko; Haga, Yoko; Hamada, Hiroko; Hamaie, Yumiko; Hamanaka, Yohei; Hanazawa, Mika; Hara, Yukari; Hasegawa, Atsushi; Hatakeyama, Asuka; Hatakeyama, Sumika; Hatanaka, Nozomi; Hatanaka, Rieko; Hidaka, Takanori; Hino, Kenji; Hirama, Hiroe; Hirano, Ikuo; Hirano, Sachiko; Hirata, Takumi; Hiratsuka, Masahiro; Hiratsuka, Yuki; Hirayama, Ikuko; Hishinuma, Eiji; Hoshi, Takako; Hozawa, Atsushi; Ido, Keisuke; Igari, Nobuko; Iida, Chikako; Imai, Katsuko; Inoue, Makiko; Inoue, Reiko; Irie, Rumi; Ishida, Motoko; Ishida, Noriko; Ishigaka, Eri; Ishii, Chihiro; Ishii, Kaori; Ishii, Osamu; Ishii, Tadashi; Ishikawa, Tatsuro; Ishikuro, Mami; Ishimori, Kazutoshi; Itabashi, Miho; Ito, Kumiko; Ito, Maiko; Ito, Masumi; Ito, Mayumi; Ito, Megumi; Ito, Natsuko; Ito, Rie; Ito, Saori; Iwabuchi, Fumihiko; Iwabuchi, Maki; Izumi, Yoko; Izumi, Yoshiko; Kambe, Masataka; Kanno, Takanari; Kano, Mayu; Kasahara, Naoko; Kashiwa, Hinako; Katahira, Kiyomi; Kato, Mayumi; Kato, Yukie; Katsuoka, Fumiki; Kawabata, Takeshi; Kawada, Rika; Kawagoe, Aoi; Kawame, Hiroshi; Kawashima, Junko; Kawashima, Yukako; Kikuchi, Junko; Kikukawa, Tsuyoshi; Kikuya, Masahiro; Kimura, Masae; Kimura, Michiko; Kinoshita, Kengo; Kishi, Ikuko; Kishimoto, Tomoko; Kitaura, Tamie; Kobayashi, Mika; Kobayashi, Tadao; Kobayashi, Tomoko; Kodama, Eiichi N.; Kodate, Shun; Kogure, Mana; Kohagizawa, Toshisada; Kohketsu, Naomi; Koida, Noa; Koide, Chie; Koide, Mika; Koike, Toshihiko; Kojima, Kaname; Komatsu, Junko; Kondo, Ayumi; Konno, Riyo; Konno, Yukie; Koreeda, Sachie; Koshiba, Seizo; Koyama, Takuya; Kudo, Hisaaki; Kumada, Kazuki; Kumadaki, Ryoko; Kumagai, Rika; Kumagai, Toshie; Kumagai, Yuko; Kunii, Yasuto; Kuriki, Miho; Kuriyama, Shinichi; Kurokawa, Emiko; Kurota, Seiko; Kusano, Hisako; Li, Bin; Li, Donghan; Li, Xue; Maeshibu, Kanako; Maeta, Keiko; Makino, Satoshi; Matsubara, Hiroko; Matsukawa, Naomi; Matsumoto, Masako; Matsuoka, Takako; Matsushita, Yuka; Matsuzaki, Motomichi; Metoki, Hirohito; Minakawa, Sayaka; Minami, Yuki; Mirei Suzuki; Mitate, Kyoko; Mito, Satomi; Miura, Ayako; Miura, Noriko; Miyagi, Ryo; Miyazawa, Akiko; Mizuno, Satoshi; Mochida, Akiko; Momii, Mika; Mori, Hiroko; Mori, Naoko; Motohashi, Hozumi; Motoike, Ikuko N.; Mugikura, Shunji; Murakami, Keiko; Murakami, Takahisa; Nagai, Masato; Nagaie, Satoshi; Nagami, Fuji; Naganuma, Toko; Nagasaka, Tatsuo; Nagase, Sachiko; Nakagawa, Kumiko; Nakai, Taku; Nakajo, Noriko; Nakamichi, Kyoko; Nakamura, Chie; Nakamura, Naoki; Nakamura, Tomohiro; Nakasato, Yuko; Nakaya, Kumi; Nakaya, Naoki; Nanatani, Kei; Narita, Akira; Narita, Yuka; Nemoto, Yasuhisa; Nishi, Hafumi; Nishida, Kohji; Nishijima, Ichiko; Nishiyama, Momo; Nobukuni, Takahiro; Nochioka, Kotaro; Noda, Aoi; Noguchi, Kenichi; Nozoe, Kiriko; Nunokawa, Rie; Obara, Taku; Obara, Tomoko; Ogasawara, Kaori; Ogawa, Satoru; Ogishima, Soichi; Oguma, Natsuki; Ohi, Nahoko; Ohisa, Namiko; Ohneda, Kinuko; Ohori, Hayami; Oikawa, Miri; Oikawa, Yumi; Ojima, Yumiko; Okada, Yumi; Okamura, Yasunobu; Okuda, Hiroshi; Okuda, Mitsuko; Okumoto, Ayako; Ono, Akane; Ono, Chiaki; Onodera, Genki; Onodera, Kaname; Onodera, Masako; Onuma, Midori; Onuma, Tomomi; Oohashi, Keiichiro; Oomachi, Masumi; Ootomo, Kazuya; Oouchi, Yukie; Orui, Masatsugu; Osada, Mayumi; Osanai, Tamae; Ota, Reiko; Otake, Noriko; Otomo, Sumie; Otsuki, Akihito; Otsuki, Yoko; Oyama, Yuki; Oyamada, Keiko; Ozawa, Yoko; Obara, Satomi; Saigusa, Daisuke; Saito, Asami; Saito, Hisako; Saito, Kazue; Saito, Manami; Saito, Megumi; Saito, Ritsumi; Saito, Sakae; Saito, Tomo; Saito, Yoko; Saito, Yuki; Saitoh, Yoshinobu; Sakai, Hiroko; Sakaida, Masaki; Sakamono, Hiroshi; Sakamoto, Hiromi; Sakamoto, Kana; Sakamoto, Mia; Sakurai, Kasumi; Sakurai, Miyuki; Sakurai, Rieko; Sakurai‐Yageta, Mika; Sasaki, Kana; Sasaki, Miho; Sasaki, Tadashi; Sasaki, Yukari; Sasaki, Yukie; Sato, Chika; Sato, Hirokazu; Sato, Michiyo; Sato, Miho; Sato, Mitsuharu; Sato, Miu; Sato, Naoko; Sato, Reiko; Sato, Satoshi; Sato, Shiho; Sato, Taku; Sato, Yoshiko; Sato, Youko; Sato, Yui; Satoh, Michihiro; Sekiya, Ayako; Seo, Mariko; Shima, Yoshiko; Shimada, Muneaki; Shimizu, Atsushi; Shimizu, Ritsuko; Shinoda, Genki; Shirakawa, Nobuyuki; Shirota, Matsuyuki; Shoji, Hiroe; Shoji, Ikuko; Shoji, Mariko; Shoji, Midori; Shoji, Wakako; Someya, Satomi; Sonobe, Shinya; Sou, Itsumi; Suenaga, Rie; Suenaga, Yasuko; Suga, Mayumi; Sugai, Rika; Sugawara, Junichi; Sugawara, Megumi; Sugawara, Michiko; Sugawara, Nanako; Sugawara, Saori; Sugawara, Yuki; Sugimoto, Sachiyo; Suzuki, Airi; Suzuki, Ayano; Suzuki, Keiko P.; Suzuki, Michirou; Suzuki, Mikiko; Suzuki, Norio; Suzuki, Rie; Suzuki, Ryoko; Suzuki, Takafumi; Suzuki, Tatsuya; Suzuki, Yoichi; Tadaka, Shu; Taguchi, Keiko; Taiji, Nozomi; Taira, Makiko; Takagi, Kaori; Takahashi, Emi; Takahashi, Harumi; Takahashi, Junko; Takahashi, Megumi; Takahashi, Noriko; Takahashi, Rieko; Takahashi, Yukiko; Takasawa, Mayuko; Takase, Masato; Takayama, Jun; Takeuchi, Miho; Takita, Sayaka; Tamahara, Toru; Tamiya, Gen; Tamura, Naomi; Tanaka, Akari; Tanaka, Saiko; Tanno, Chihiro; Tanno, Naoko; Tateno, Keiko; Tateno, Minoru; Terui, Chika; Toki, Mihoko; Tokioka, Sayuri; Tomita, Etsuko; Tomita, Hiroaki; Tomizuka, Mai; Tsuchiya, Naho; Tsuda, Miyuki; Tsumuraya, Tomomi; Tsunasawa, Junko; Tsunoda, Issei; Uchiya, Juri; Ueda, Akiko; Ueki, Yuriko; Ueno, Fumihiko; Umeda, Keiko; Uruno, Akira; Wada, Ikuko; Wada, Tomoko; Wagatsuma, Mika; Watanabe, Hitoshi; Watanabe, Kanako; Watanabe, Kazue; Yaegashi, Nobuo; Yagyu, Mika; Yamada, Etsuko; Yamaguchi; Kabata, Yumi; Yamamoto, Hiroko; Yamamoto, Masayuki; Yamauchi, Yukari; Yamazaki, Mika; Yasuda, Jun; Yin, Hang; Yokota, Hiroshi; Yokoyama, Manami; Yokoyama, Marie; Yokoyama, Tomoko; Yoshida, Yuko; Yoshino, Mizue; Yu, Zhiqian; Zhang, Lin.

## CONFLICT OF INTEREST STATEMENT

The authors declare no conflicts of interest.

## ETHICAL APPROVAL STATEMENT

This study was approved by the ethics committee of the Tohoku University School of Medicine (approval: 2013‐4‐103; date: May 10, 2013; latest revision: 2024‐4‐021; approval date: April 22, 2024). This study followed the consensus framework for ethical standards, applicable laws, and regulations. The study was authorized by the Institutional Review Boards and Independent Ethics Committees.

## PATIENT CONSENT STATEMENT

N/A.

## CLINICAL TRIAL REGISTRATION

N/A.

## Supporting information

Supporting information.

## Data Availability

All data used to support the findings may be released upon application to the Tohoku Medical Megabank Organization.
